# Health Claims, Product Features and Instructions for Use on the Labels of Potassium-enriched Salt Products: A Content Analysis

**DOI:** 10.1016/j.cdnut.2024.104473

**Published:** 2024-10-09

**Authors:** James Bullen, Xuejun Yin, Katrina Kissock, Laura Fisher, Bruce Neal, Kathy Trieu

**Affiliations:** 1 The George Institute for Global Health, University of New South Wales, New South Wales, Australia; 2 School of Population Medicine and Public Health, Chinese Academy of Medical Sciences and Peking Union Medical College, Beijing, China; 3 School of Public Health, Imperial College, London, United Kingdom

**Keywords:** sodium chloride, sodium reduction, potassium-enriched salt, low-sodium salt, salt substitute, potassium chloride, cardiovascular disease, blood pressure, content analysis, labeling

## Abstract

**Background:**

Potassium-enriched salt is a proven dietary intervention for reducing risk of stroke, cardiovascular disease, and premature mortality when used instead of regular table salt. Potassium-enriched salt products are available globally, but the on-pack health claims, product features, and instructions for use are diverse.

**Objectives:**

The objective of this study was to summarize the label features of potassium-enriched salt products available worldwide.

**Methods:**

A content analysis was conducted on the labels of potassium-enriched salt products available for sale in May 2023. Potassium-enriched salt products were identified through a systematic search of literature, major online shopping websites, and Google using similar keywords such as “salt substitute” or “low sodium salt.” Information on product labels was coded relating to health claims, product features, and instructions for use, and were quantitatively summarized.

**Results:**

A total of 117 potential potassium-enriched salt products were identified, with 83 included in the final analysis after excluding products containing no sodium (*n* = 15), no potassium (*n* = 5), or that were duplicates (*n* = 14). There were 23 (28%) products with on-pack claims for health benefits and 36 (43%) with health warnings. Twenty-five (30%) of product labels included descriptions of other product features such as taste and potassium content, and 36 (43%) had instructions for use.

**Conclusions:**

There was large variability in the label features of potassium-enriched salt products identified in this study. Most product labels made no health-related statements, but among those that did, warnings occurred more frequently than statements of health benefits. The diversity in labeling may cause confusion among consumers, and standardized, evidence-based labeling should be developed.

## Introduction

Cardiovascular diseases are the leading cause of death worldwide, and high blood pressure is a primary underlying driver of cardiovascular disease risk [1]. Overconsumption of dietary sodium and underconsumption of dietary potassium both raise blood pressure, leading to increased rates of stroke, heart attack, heart failure, kidney disease, and early death [2]. The WHO set a target of a 30% reduction in population sodium intake by 2025, with 194 member states of the United Nations having adopted this recommendation, though none are set to meet the target [[3], [4], [5]]. The WHO also provides a strong recommendation for increasing the population’s potassium intake levels [6].

To achieve sodium reduction goals, the WHO has identified 4 strategies it considers “best-buy” interventions [7]. These interventions include reformulation targets for sodium content in foods, public food procurement policies that limit foods high in sodium in public institutions, front-of-package labeling to help consumers choose products lower in sodium, and mass media campaigns to change behaviors and reduce sodium consumption. The WHO also strongly advocates for the consumption of more fresh fruits and vegetables to increase dietary potassium intake [6]. Despite these recommendations and numerous attempts at interventions, sodium consumption around the world remains high, with only isolated instances of success [4]. Reducing discretionary sodium intake has been particularly difficult because it requires people to change cooking and seasoning behaviors and accept a different taste [5]. There has been a similar lack of progress with increasing consumption of fresh fruits and vegetables and increasing dietary potassium intake with the cost of fruits and vegetables, availability of these products, and time required to purchase and prepare all identified as significant barriers to increased consumption [[8], [9], [10]].

Potassium-enriched salt (a type of salt substitute) is a dietary intervention that jointly reduces sodium intake and increases potassium intake. Strong evidence that potassium-enriched salt reduces blood pressure has existed for over a decade [[11], [12], [13]]. Large-scale clinical trials and reviews show that these products also reduce risks of strokes, major cardiovascular events, and premature mortality [11,14,15] across a range of geographical locations, including Brazil, France, India, Finland, the United Kingdom, Denmark, and China. Studies have been conducted among normotensive and hypertensive people and involve the replacement of regular table salt use with potassium-enriched salt. However, the majority of such trials are in China and among older people with high blood pressure. The totality of the relevant data suggests that the benefits of potassium-enriched salt are likely to be generalizable to large populations across the globe and that the intervention is likely to be highly cost-effective [11,16]. Another key observation is that switching to potassium-enriched salt appears to be feasible and sustainable, with the largest randomized controlled trial conducted in China, finding 92% of >10,000 intervention group participants assigned to potassium-enriched salt continuing to use it after 5 y [14]. Key reasons for continued use appear to have been that *switching* salt use is easier than *cutting* salt use because cooking and seasoning behaviors do not need to change, and there is no substantive change in taste [17].

Although the use of potassium-enriched salt is likely to be beneficial and safe for most of the world’s population, it is contraindicated in people with advanced kidney disease and people using potassium supplements or potassium-sparing diuretics because of the risk of hyperkalemia (excessively high blood potassium concentrations) [18]. Some clinical guidelines provide specific recommendations for use, but potassium-enriched salt products should also have accurate data on packs relating to health claims, health warnings, instructions for use, and content [[19], [20], [21], [22]]. Product labeling can also have an important influence on purchasing behaviors [[23], [24], [25]].

With an expected increase in the use of potassium-enriched salt around the world, given the growing evidence base and call for clinical guidelines to systematically recommend its use, it is important that product labels accurately declare the potential health benefits and risks and describe how potassium-enriched salt should be used [[26], [27], [28], [29], [30]]. The aim of this study was to systematically summarize the information currently provided on the packaging of potassium-enriched salt products available for sale around the world.

## Methods

We used a content analysis approach adapted from Conway et al. [31] (2023), whereby we first searched for potassium-enriched salt products available globally and then characterized and analyzed the text on product labels. Ethics committee approval was not required nor sought for this project.

### Identification of products

We identified potassium-enriched salt products firstly using a prior database of products that the author (XY) compiled in 2020 through a search of peer-reviewed literature, a search of major online shopping sites, a keyword search in 6 languages using the Google search engine, and a consultation with global experts in sodium reduction [26]. The details of the database search strategy are published previously [26]. The lead author (JB) then repeated the searches of the literature, online shopping sites, and Google in May 2023 in English to capture more recent products (see Supplemental Material for more detail and the search terms).

### Inclusion and exclusion criteria

Eligible potassium-enriched salt products were those for human consumption that had a proportion of sodium chloride content replaced with potassium chloride. We excluded products that did not contain sodium or potassium in their nutrition information panel or ingredients list (e.g., herb salts and products that were 100% potassium chloride or 100% sodium chloride). We also excluded duplicate products, which were defined as products with identical sodium chloride and potassium chloride and product names sold in a country outside the product’s country of manufacture. Products were screened for inclusion and exclusion criteria by 1 author (JB).

### Data extraction and coding

The task of extraction of the label content was shared between 2 researchers (XY and JB). Label content was extracted into a predesigned Excel spreadsheet. Data sought from each product label included the product name, brand, country of manufacture, and formulation (including quantitative sodium and potassium content and other substantive inclusions of magnesium, calcium, or iodine). All written text on the front and back of the product label, except for some nutritional values on the product’s nutrition information panel that were outside the scope of this manuscript, were also extracted and reviewed. Two researchers (JB and XY) independently categorized the text on the label of eligible products into 4 broad categories based on previous research [26]:

*1*) Health benefits - defined as wording that describes a positive actual or potential effect of the consumption of the product on specific health outcomes.
*2*) Health warnings - defined as wording that describes a negative actual or potential effect or consequence of the consumption of the product on health outcomes or that recommends against consumption of the product in some way or for some group of people.
*3*) Instructions for use - defined as any wording about how the product should or should not be used. This included recommendations or advice to consult a medical professional before consuming the product.
*4*) Product content - defined as any wording about the product’s intrinsic qualities or characteristics. This included nutrition content claims about the presence, absence, reduction, or fortification of a vitamin, mineral, or other substance in the product, as well as content relating to the flavor of these products.

### Analysis

Using a deductive coding approach, the 2 researchers (JB and XY) identified and coded text to themes nested under the 4 broad categories described above. These themes captured common ideas or concepts on the text of labels (such as the specific health benefits related to blood pressure or muscle function) and were refined through discussion between the researchers [32,33]. Coder 1 (JB) performed initial coding, whereas Coder 2 (XY) re-coded 10% of the data to check for accuracy. Once the text was coded, we analyzed codes quantitatively using both the number of products and the total number of coding instances as the denominator reporting frequencies and proportions [32,34]. In addition, we assigned the country’s income level and region based on the World Bank classification [35]. This was done to help contextualize the findings against other relevant observations, such as the association between country income and the proportion of dietary sodium that comes from discretionary salt. We explored product label information by country for countries with >2 products and sought to identify patterns in the claims made for products that had >1 claim on the label. Organization, coding, and analysis of the data were performed in the research software NVivo (release 1.7.1) and with R (version 4.3.1) and RStudio (“Desert Sunflower” 2023.09.0+463 release) [[36], [37], [38]].

## Results

**FIGURE 1:**
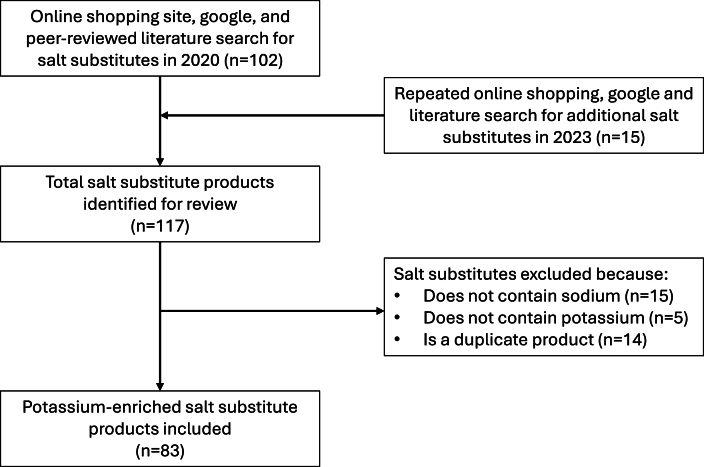
Flow diagram for the inclusion of potassium-enriched salt products.

A total of 117 potential potassium-enriched salt products were identified, with 83 included in the final analysis after excluding products containing no sodium (*n* = 15), no potassium (*n* = 5), or that were duplicates (*n* = 14) (Figure 1). The included products were manufactured in 39 countries. China produced the highest number of potassium-enriched salt products (*n* = 21), followed by the United States (*n* = 9) and India (*n* = 4). Among the products identified, 10 have ≤15% potassium chloride, 19 have 16–30% potassium chloride, 16 have 31–50% potassium chloride, and 6 contain >50% potassium chloride (Table 1) [35].

**TABLE 1:** Characteristics of included potassium-enriched salt products.

Characteristics	Number of products, *n* (%) (Total = 83)
Region^1^	
East Asia and Pacific	34 (41%)
Europe and Central Asia	22 (27%)
North America	10 (12%)
Latin America and Caribbean	5 (6%)
Middle East and North Africa	5 (6%)
South Asia	5 (6%)
Sub-Saharan Africa	2 (2%)
Country income level^1^	
Lower-middle-income	8 (10%)
Upper-middle-income	36 (43%)
High-income	39 (47%)
Proportion of sodium chloride (%)	
≤15	1 (1%)
16–30	3 (4%)
31–50	28 (37%)
51–70	19 (25%)
>70	25 (33%)
Proportion of potassium chloride (%)	
≤15	10 (20%)
16–30	19 (37%)
31–50	16 (31%)
51–70	5 (10%)
>70	1 (2%)

1 Region and country income level as defined by the World Bank [35].

Among the 83 products, 55 (66%) had labels with text describing benefits, warnings, instructions, or content. The highest number of discrete label features on any 1 product was 8, and there were 28 (34%) products with no claim (Table 2).

**TABLE 2:** Number of potassium-enriched products with label features and the total number of label features used.

Category		Products *n*, (%) (Total = 83)	Label features *n*, (%) (Total = 205)
Health benefits		23 (28)	33 (16)
Health warnings		36 (43)	88 (43)
Instructions for use		36 (43)	39 (19)
Product content		25 (30)	45 (22)

### Health benefits

There were 23 (28%) products with labels containing health benefit information. The most common health benefit claims were that products “help manage blood pressure” (*n* = 7), “help maintain blood pressure” (*n* = 7), are beneficial to heart health, or “promote a healthy heart” (*n* = 6), or help reduce sodium intake (*n* = 5) (Table 3). Products also made claims about being a “healthy choice,” “healthy alternative,” or “part of a healthy lifestyle” (*n* = 11). Several other health claims were made less commonly, including that a potassium-enriched salt helps to control weight (*n* = 1) or that it reduces fluid retention (*n* = 1).

**TABLE 3:** Label features on potassium-enriched salt products.

	Products, *n* (%) Total = 83
Health benefits	
Low-sodium products or diets help manage blood pressure	7 (3)
Helps maintain blood pressure	7 (3)
Helps with heart health or promotes a healthy heart	6 (3)
Helps reduce sodium intake	5(2)
Helps with potassium intake	3 (2)
Calcium protection	1 (1)
Contributes to the normal functioning of muscles	1 (1)
Helps control weight	1 (1)
Health choice, healthy alternative, or part of a healthy lifestyle	11 (5)
Health warnings	
Avoid if you have renal dysfunction, kidney disorders, or disease	25 (12)
Avoid taking antihypertensive drugs	17 (8)
Avoid in high-temperature or if a worker is doing heavy labor	17 (8)
Avoid if on a low potassium or potassium-restricted diet	12 (6)
Avoid if on a low sodium or sodium-restricted diet	6 (3)
Avoid if you have heart failure or heart problems	5 (2)
Avoid if you have diabetes	2 (1)
Avoid if you have high blood pressure	2 (1)
Avoid if you have hyperthyroidism or thyroid disease	2 (1)
Not suitable for use with certain diuretics	2 (1)
Instructions for use	
Use with caution	17 (8)
Consult with a doctor before use	14 (7)
Do not use unless approved by a doctor	3 (1)
Use under the supervision of a physician	2 (1)
Use sparingly	1 (1)
Product content	
Less sodium is lower sodium, or is sodium-reduced	13 (6)
Flavorful or tasty	9 (4)
Contains potassium chloride	7 (3)
A good source of or enriched with potassium	2 (1)
Contains or is enriched with magnesium	1 (1)
Contains or is enriched with selenium	1 (1)

### Health warnings

Thirty-six (43%) products had labels with health warnings that mostly identified a group or groups of people who should not use the product. The most commonly occurring warnings were related to people with “renal dysfunction,” “kidney disorders,” or “kidney concerns” (*n* = 25) (Table 3), with 17 products produced by the China Salt Corporation appearing to have a standardized warning about “renal dysfunction.” Other common “avoidance” warnings were for patients with hypertension taking antihypertensive drugs (*n* = 17) or for workers in high-temperature settings or doing heavy labor (*n* = 17). Both warnings were heavily represented because they appeared to be a part of a standardized label used for the 17 China Salt Corporation products.

### Instructions for use

Thirty-six (43%) products had labels with instructions for use. Products were most often labeled with instructions to “use with caution” (*n* = 17) or to “consult with a doctor before use” (*n* = 14) (Table 3). Less common was the more strongly worded instruction not to use the product “unless approved by a doctor” (*n* = 3), not to use with certain diuretics (*n* = 2), or to use only under physician supervision (*n* = 2). Instructions to consult with a doctor or use with caution were usually specific in nature, applying to groups of people such as those requiring diets low in sodium or potassium or those on medication for heart disease, diabetes, or kidney diseases. Some labels, however, made a general recommendation to seek a doctor’s advice before use.

### Product content

Twenty-five (30%) products had labels with text relating to content, most frequently “less sodium,” “is lower sodium,” or “is sodium-reduced” (*n* = 13) or of being flavorful or tasty (*n* = 9). Nine products included claims about potassium chloride content either by simply stating that the product contains potassium chloride (*n* = 7) or “is a good source of” or “is enriched with” potassium chloride (*n* = 2).

### Patterns of claims across products

Six countries had >2 potassium-enriched salt products: China (*n* = 21), the United States (*n* = 9), India (*n* = 4), Russia (*n* = 3), Singapore (*n* = 3) and the United Kingdom (*n* = 3). In general, product labels in these countries tended toward warnings and not claims of benefits. Products in China showed a degree of standardization in labeling that was not observed in other countries due to a national standard that regulates label content and which manufacturers must follow [39]. The labels in China mentioned health benefits less frequently than in other countries (10% compared with 34%) and health warnings more frequently than in other countries (81% compared with 31%). Some texts covered >1 category of claim, such as “people with heart or kidney problems should consult their doctor before use,” which is both a warning and an instruction for use. There were otherwise no discernible patterns in how claims appeared on products when multiple claims were made on a single product.

## Discussion

There was large variability in the label features of potassium-enriched salt products identified in this study. The majority of product labels made no health-related statements at all, but among those that did, warnings occurred more frequently than statements of health benefits. The diversity in labeling may cause confusion among consumers, and the potential for standardized labeling should be investigated further. Product content claims were more likely to be about lower sodium content than higher potassium content, but most made no such content claim. Seventeen products from China showed some standardized labeling focused on warnings with all products manufactured by the China Salt Corporation [40]. There are many potassium-enriched salt products available worldwide, but it appears that most consumers who consider purchasing these products are getting incomplete and potentially misleading information about the product’s suitability for their needs.

There is a strong case for every potassium-enriched salt product to carry a claim about the potential health benefit of using the salt and that such a claim is comparable across markets. Evidence suggests that product labels and claims made on products influence consumer purchases and that giving consumers specific, evidence-based information can support choices of products beneficial to their health [41,42]. There is limited research on products that contain both claims of health benefits and health warnings, with most studies in this area focused on labeling unhealthy foods, though a recent study found that people exposed to both positive and negative food labels made healthier selections than those exposed to just 1 or the other [43]. There is very clear evidence that the use of potassium-enriched salt as a replacement for discretionary salt will lower blood pressure and reduce the risks of stroke and other cardiovascular diseases in people with hypertension [11,14,44]. Products without claims and products with nonspecific claims, such as a “healthy choice” or “good for a healthy heart,” might instead carry more specific wording related to potassium-enriched salt reducing blood pressure when used as a replacement for table salt and cooking salt. In some regulatory contexts, the process of placing a health claim on the package can be intensive or challenging – such as in Australia and New Zealand, where health claims linking a nutrient or substance in a food with a specific biomarker of disease (e.g., blood pressure) require an application to the food regulator, amendment of the food standards code, and significant costs for the applicant [45]. This may explain some of the current gaps between known evidence and on-package claims.

It is also clear that potassium-enriched salt products should carry a warning about potential harm. Although no trial of potassium-enriched salt has shown any harm, at-risk patients (such as those using a potassium-sparing diuretic, potassium supplement, or with known serious kidney disease) have mostly been excluded from those studies [11]. There is, however, no evidence and little physiologic rationale to support warnings against use outside of these patient groups. In this analysis, we found some warnings on products that are not supported by evidence, such as instructing people to avoid them when doing heavy labor if they work in high temperatures, have high blood pressure, or have thyroid disease.

Regardless of the exact form of benefits claimed or health warning used, it is vital that all products include both so that consumers can make fully informed decisions. Large numbers of people worldwide stand to benefit from switching to potassium-enriched salt from regular salt, especially in countries where discretionary salt use accounts for more than half of sodium consumption and where the burden of cardiovascular disease is high (such as China, India, and Brazil) [[46], [47], [48]]. Very few of the population are at risk of hyperkalemia, and although it is important to protect them, it is equally important to inform the many more who could benefit from switching to potassium-enriched salt. It is likely that manufacturers and retailers of potassium-enriched salt are uncertain about the optimum form of wording since few would have the expertise to synthesize and interpret all applicable data. Submission of proposed labeling for potassium-enriched salt to credentialled national food regulators, with supporting documents that summarize the evidence base, is 1 mechanism proposed for securing standardized wording that properly expresses risks and benefits for consumers. The use of available reviews of evidence and expert panel agreement to develop a standardized label should be a priority. A standardized label could highlight evidence-based knowledge of the health effects of switching to potassium-enriched salt, such as: “Potassium-enriched salt helps maintain a healthy blood pressure and can reduce the risk of stroke when used as a replacement for regular salt.” Additionally, it could recommend standardized evidence-based cautions such as: “If you have been told to limit potassium in your diet, consult your doctor or other healthcare professional before use.” Such labels could also make clear that the product is intended to be used as a replacement, not an addition, for regular table salt use. This potential wording is preliminary in nature. We emphasize that it is based on current findings only and a full evidence review and expert consultation is needed prior to adoption.

The instructions for use were mostly related to managing the risk of hyperkalemia. Instructions might also usefully indicate that the potassium-enriched salt should be used as a direct switch for regular salt without the need for any other change since this is likely to be important to uptake. In regard to content claims, it may be helpful for products to systematically highlight the additional potassium content since “potassium enrichment” seems more likely to elicit favorable responses than “reduced sodium,” which is widely associated with less or worse taste [49,50].

A better understanding of the policy environment and regulatory frameworks controlling product labeling and claims would be useful in understanding why certain content appears on product labels, as well as the scope for changing that content. In particular, understanding how regulatory frameworks were used to make claims clearly unsupported by the scientific literature, such as the claim that potassium-enriched salt helps to control weight, would be a priority. There may also be an opportunity to engage with potassium-enriched salt manufacturers and other industry stakeholders to standardize claims made on products so that consumers are properly informed and their interests are adequately protected.

A key strength of this report is the inclusion of a large number of products from diverse countries and manufacturers. Although we may not have captured all potassium-enriched salt products available on the global market, given that the updated search in 2023 was only conducted in English, and some own-brand products may be missed, it is likely we have captured the most commonly purchased potassium-enriched salts. An analysis specific to the Chinese market, though not restricted solely to potassium-enriched salt products, found a higher number of low-sodium salts in China [51] and was also focused on the text on the product labels. Our analysis did not address the graphics on the product packaging or associated online marketing material found on web pages and did not search all online marketplaces. We noted that some products contained graphical elements that could convey meaning or implications about the product. For example, an image of a heart could imply to consumers that the product is good for heart health. Likewise, we did not analyze marketing information on product webpages, and future investigations comparing the health claims made using off-pack promotion methods would be a useful extension of our work.

In conclusion, the study identified a range of labeling claims and features among potassium-enriched salt products. Most (>50%) products did not include health claims or warnings. Of those that did, labels tended to highlight health warnings or advised caution rather than promoting health benefits or other positive aspects of the products. Claims regarding product content were more often focused on reduced-sodium concentrations rather than increased potassium concentrations. There is a clear need and significant opportunity to provide standardized, evidence-based product information on potassium-enriched salt that effectively communicates to consumers both the potential health benefits and potential risks of switching use. Given the strong body of evidence, the label should highlight the benefits of potassium-enriched salt in lowering blood pressure and risk of stroke and make clear that such benefits relate to the use of the salt as a replacement for regular table salt. In addition, the label should instruct those told to limit potassium intake to consult their doctor or healthcare professional before using the potassium-enriched salt to help mitigate potential risks of hyperkalemia. Improvements in the information provided on potassium-enriched salt products based on well-established evidence are urgently needed to support its appropriate use.

## Author contributions

The authors’ responsibilities were as follows – JB, XY, KT: designed the research; JB, XY: conducted the research; JB: analyzed the data; JB, XY, KT: wrote the article; JB: had primary responsibility for final content. All authors provided critical feedback on the manuscript; and all authors: read and approved the final manuscript.

## Conflict of interest

The authors report no conflicts of interest.

## Funding

The authors reported no funding received for this study.

## Data availability

The data set supporting the conclusions of this article is included within the article and its additional file.

## References

[1] Mensah G.A., Roth G.A., Fuster V. (2019). The global burden of cardiovascular diseases and risk factors: 2020 and beyond. J Am. Coll. Cardiol..

[2] Huang L., Trieu K., Yoshimura S., Neal B., Woodward M., Campbell N.R. (2020). Effect of dose and duration of reduction in dietary sodium on blood pressure levels: systematic review and meta-analysis of randomised trials. BMJ.

[3] World Health Organization (2014).

[4] World Health Organization (2023).

[5] Santos J.A., Tekle D., Rosewarne E., Flexner N., Cobb L., Al-Jawaldeh A. (2021). A systematic review of salt reduction initiatives around the world: a midterm evaluation of progress towards the 2025 global non-communicable diseases salt reduction target. Adv. Nutr..

[6] World Health Organization (2012). Guideline: potassium intake for adults and children.

[7] World Health Organization (2017). Tackling NCDs: 'best buys' and other recommended interventions for the prevention and control of noncommunicable diseases.

[8] Reddin C., Ferguson J., Murphy R., Clarke A., Judge C., Griffith V. (2023). Global mean potassium intake: a systematic review and Bayesian meta-analysis. Eur. J Nutr..

[9] Kalmpourtzidou A., Eilander A., Talsma E.F. (2020). Global vegetable intake and supply compared to recommendations: A systematic review. Nutrients.

[10] Livingstone K.M., Burton M., Brown A.K., McNaughton S.A. (2020). Exploring barriers to meeting recommendations for fruit and vegetable intake among adults in regional areas: A mixed-methods analysis of variations across socio-demographics. Appetite.

[11] Yin X., Rodgers A., Perkovic A., Huang L., Li K.C., Yu J. (2022). Effects of salt substitutes on clinical outcomes: a systematic review and meta-analysis. Heart.

[12] Hernandez A.V., Emonds E.E., Chen B.A., Zavala-Loayza A.J., Thota P., Pasupuleti V. (2019). Effect of low-sodium salt substitutes on blood pressure, detected hypertension, stroke and mortality. Heart.

[13] China Salt Substitute Study Collaborative Group (2007). Salt substitution: a low-cost strategy for blood pressure control among rural Chinese. A randomized, controlled trial. J Hypertens.

[14] Neal B., Wu Y., Feng X., Zhang R., Zhang Y., Shi J. (2021). Effect of salt substitution on cardiovascular events and death, N Engl. J Med..

[15] Yuan Y., Jin A., Neal B., Feng X., Qiao Q., Wang H. (2023). Salt substitution and salt-supply restriction for lowering blood pressure in elderly care facilities: A cluster-randomized trial. Nat Med.

[16] Li K.C., Huang L., Tian M., Di Tanna G.L., Yu J., Zhang X. (2022). Cost-effectiveness of a household salt substitution intervention: findings from 20 995 participants of the salt substitute and stroke study. Circulation.

[17] Li N., Prescott J., Wu Y., Barzi F., Yu X., Zhao L. (2009). The effects of a reduced-sodium, high-potassium salt substitute on food taste and acceptability in rural northern China, Br. J Nutr.

[18] Yin X., Tian M., Sun L., Webster J., Trieu K., Huffman M.D. (2021). Barriers and facilitators to implementing reduced-sodium salts as a population-level intervention: A qualitative study. Nutrients.

[19] Ide N., Ajenikoko A., Steele L., Cohn J., Curtis C.J., Frieden T.R. (2020). Priority actions to advance population sodium reduction. Nutrients.

[20] Wang T.D., Chiang C.E., Chao T.H., Cheng H.M., Wu Y.W., Wu Y.J. (2022). 2022 guidelines of the Taiwan Society of Cardiology and the Taiwan Hypertension Society for the Management of Hypertension, Acta. Cardiol. Sin..

[21] (2019). Joint Committee for Guideline Revision, 2018 Chinese guidelines for prevention and treatment of hypertension-A report of the revision committee of Chinese guidelines for prevention and treatment of hypertension. J Geriatr. Cardiol..

[22] Williams B., Mancia G., Spiering W., Agabiti Rosei E., Azizi M., Burnier M. (2018). 2018 ESC/ESH Guidelines for the management of arterial hypertension. Eur. Heart J..

[23] Pinjuh Markota N.P., Rumboldt M., Rumboldt Z. (2015). Emphasized warning reduces salt intake: a randomized controlled trial. J Am. Soc. Hypertens..

[24] Maganja D., Buckett K., Stevens C., Flynn E. (2019). Consumer choice and the role of front-of-pack labelling: the Health Star Rating system, Public Health Res. Pract..

[25] da Silva C.P., Bento A.C., Guaraldo E. (2022). The impact of front-of-the-packaging nutrition labelling warnings on consumer habits: a scoping review exploring the case of the Chilean Food Law. Br. Food J..

[26] Yin X., Liu H., Webster J., Trieu K., Huffman M.D., Miranda J.J. (2021). Availability, formulation, labeling, and price of low-sodium salt worldwide: environmental scan. JMIR Public Health Surveill.

[27] Jones D.W., Clark D., Morgan T.O., He F.J. (2022). Potassium-enriched salt substitution as a population strategy to prevent cardiovascular disease. Hypertension.

[28] Yin X., Paige E., Tian M., Li Q., Huang L., Yu J. (2023). The proportion of dietary salt replaced with potassium-enriched salt in the SSaSS: implications for scale-up. Hypertension.

[29] Kissock K.R., Garrett G.S., Mkambula P., Bullen J.D., Trieu K., Fisher L.J. (2024). Switching the world’s salt supply – learning from iodization to achieve potassium enrichment. Adv. Nutr..

[30] Xu X., Zeng L., Jha V., Cobb L.K., Shibuya K., Appel L.J. (2024). Potassium-enriched salt substitutes: a review of recommendations in clinical management guidelines. Hypertension.

[31] Conway R., Esser S., Steptoe A., Smith A.D., Llewellyn C. (2023). Content analysis of on-package formula labelling in Great Britain: use of marketing messages on infant, follow-on, growing-up and specialist formula. Public Health Nutr.

[32] Kondracki N.L., Wellman N.S., Amundson D.R. (2002). Content analysis: review of methods and their applications in nutrition education. J Nutr. Educ. Behav..

[33] Hsieh H.F., Shannon S.E. (2005). Three approaches to qualitative content analysis. Qual. Health Res..

[34] Kaefer F., Roper J., Sinha P.N. (2015). A software-assisted qualitative content analysis of news articles: examples and reflections. Forum Qual. Soc. Res..

[35] World Bank (2020).

[36] R Core Team (2013).

[37] RStudio Team (2022).

[38] Lumivero. NVivo (version 14). Lumivero; 2023.

[39] The Standardization Administration of the People's Republic of China (2015). GB 2721-2015 National Food Safety Standard Edible Salt.

[40] Kong B., Yang S., Long J., Tang Y., Liu Y., Ge Z. (2023). National initiatives on salt substitutes: a scoping review. JMIR Public Health Surveill.

[41] Shrestha A., Cullerton K., White K.M., Mays J., Sendall M. (2023). Impact of front-of-pack nutrition labelling in consumer understanding and use across socio-economic status: A systematic review. Appetite.

[42] Ballco P., Gracia A. (2022). Tackling nutritional and health claims to disentangle their effects on consumer food choices and behaviour: A systematic review. Food Qual. Preference..

[43] Grummon A.H., Musicus A.A., Moran A.J., Salvia M.G., Rimm E.B. (2023). Consumer reactions to positive and negative front-of-package food labels. Am. J Prev. Med..

[44] Bernabe-Ortiz A., Rosas V.G.S.Y., Ponce-Lucero V., Cárdenas M.K., Carrillo-Larco R.M., Diez-Canseco F. (2020). Effect of salt substitution on community-wide blood pressure and hypertension incidence. Nat. Med..

[45] Neale E.P., Tapsell L.C. (2022). Nutrition and health claims: consumer use and evolving regulation. Curr. Nutr. Rep..

[46] Marklund M., Singh G., Greer R., Cudhea F., Matsushita K., Micha R. (2020). Estimated population wide benefits and risks in China of lowering sodium through potassium enriched salt substitution: modelling study. BMJ.

[47] Marklund M., Tullu F., Raj Thout S., Yu J., Brady T.M., Appel L.J. (2022). Estimated benefits and risks of using a reduced-sodium, potassium-enriched salt substitute in India: A modeling study. Hypertension.

[48] Bhat S., Marklund M., Henry M.E., Appel L.J., Croft K.D., Neal B. (2020). A systematic review of the sources of dietary salt around the world. Adv. Nutr..

[49] Liem D.G., Miremadi F., Keast R.S.J. (2011). Reducing sodium in foods: the effect on flavor. Nutrients.

[50] Hoppu U., Hopia A., Pohjanheimo T., Rotola-Pukkila M., Mäkinen S., Pihlanto A. (2017). Effect of salt reduction on consumer acceptance and sensory quality of food. Foods.

[51] Meng D., Cobb L.K., Ide N., Ge Z. (2023). The availability, price, and characteristics of low sodium salt based on an online salt market survey in China: implications for scaling up its use. J Clin. Hypertens..

