# Supplementary material for: Health Claims, Product Features and Instructions for Use on the Labels of Potassium-enriched Salt Products: A Content Analysis
Source: Curr Dev Nutr. 2024 Oct 9;8(11):104473. doi: 10.1016/j.cdnut.2024.104473 (PMC11547896; doi:10.1016/j.cdnut.2024.104473)
Supplement: multimedia component 1 [file mmc1.docx]

**Manuscript title: Health claims, product features and instructions for use on the labels of potassium-enriched salt products: a content analysis**

**First author’s name: James Bullen**

**Supplementary Material**

1. Systematic literature search.

A systematic literature search was conducted in MEDLINE, Embase, and Cochrane Library from inception through May 2023.

Database: Ovid MEDLINE(R) and Embase 1946 to present, Cochrane library

1. Salt.ti,ab

2. salt substitut$.ab,ti.

3. (low sodium adj5 salt).ti,ab.

4. (sodium free adj5 salt).ti,ab.

5. salt replac$.ti,ab.

6. mineral adj5 salt.ti,ab.

7. smart salt.ti,ab.

8. sodium reduced adj5 salt.ti,ab.

9. health$ salt.ti,ab.

10. potassium adj5 salt.ti,ab.

11. potassium-rich* adj5 salt.ti,ab.

12. lite salt.ti,ab.

13. sodium adj5 potassium.ti,ab.

14. NaCl.ti,ab.

15. 1 and 13

16. 1 and 14

17. or/2-12,15,16

18. limit 17 to humans

1. Search of major online shopping sites.

Major global online shopping sites – Amazon, eBay, Walmart, JD, and RedMart – were searched to identify low-sodium salts using the following keywords:

“low-sodium salt,” “salt substitute,” “potassium salt,” “mineral salt,” “sodium reduced salt.”

1. Keyword search using Google search engine.

A search was executed using Google advanced engine with a Google Chrome browser from Australia using the following keywords:

“low-sodium salt,” “salt substitute,” “potassium salt,” “mineral salt,” “sodium reduced salt.”
